# Polycystic Ovary Syndrome and Endometrial Cancer: A Scoping Review of the Literature on Gut Microbiota

**DOI:** 10.3390/cells11193038

**Published:** 2022-09-28

**Authors:** Amog Prakash, Milad Nourianpour, Abiola Senok, William Atiomo

**Affiliations:** College of Medicine, Mohammed Bin Rashid University of Medicine and Health Sciences, Building 14, Dubai P.O. Box 505055, United Arab Emirates

**Keywords:** gut microbiota, endometrial cancer, gut dysbiosis, polycystic ovary syndrome, gut microbiome, inflammation, gut-brain axis, estrobolome

## Abstract

Gut dysbiosis has been associated with polycystic ovary syndrome (PCOS) and endometrial cancer (EC) but no studies have investigated whether gut dysbiosis may explain the increased endometrial cancer risk in polycystic ovary syndrome. The aim of this scoping review is to evaluate the extent and nature of published studies on the gut microbiota in polycystic ovary syndrome and endometrial cancer and attempt to find any similarities between the composition of the microbiota. We searched for publications ranging from the years 2016 to 2022, due to the completion date of the ‘Human Microbiome Project’ in 2016. We obtained 200 articles by inputting keywords such as ‘gut microbiome’, ‘gut microbiota’, ‘gut dysbiosis’, ‘PCOS’, and ‘endometrial cancer’ into search engines such as PubMed and Scopus. Of the 200 identified in our initial search, we included 25 articles in our final review after applying the exclusion and inclusion criteria. Although the literature is growing in this field, we did not identify enough published studies to investigate whether gut dysbiosis may explain the increased EC risk in PCOS. Within the studies identified, we were unable to identify any consistent patterns of the microbiome similarly present in studies on women with PCOS compared with women with EC. Although we found that the phylum Firmicutes was similarly decreased in women with PCOS and studies on women with EC, there was however significant variability within the studies identified making it highly likely that this may have arisen by chance. Further research pertaining to molecular and microbiological mechanisms in relation to the gut microbiome is needed to elucidate a greater understanding of its contribution to the pathophysiology of endometrial cancer in patients with polycystic ovarian syndrome.

## 1. Introduction

The human body hosts trillions of microorganisms that play a pivotal role in modulating normal physiology and immune functions that are essential to our normal functioning; this effect is mediated through the production of bi-products and metabolites [1]. The term ‘microbiome’ refers to the microbes and their collective genomes within a community, while ‘microbiota’ refers to the “assemblage of microorganisms present in a defined environment” including bacteria, fungi, or archaea, but is more frequently used for bacteria composition and it refers to the microbes themselves in aggregate [2,3]. Up to 90% of the gut microbiota consists of two phyla, namely, Firmicutes and Bacteroidetes. The remaining 10% includes Actinobacteria, Proteobacteria, Fusobacteriota, and Verrucomicrobia [4].

Polycystic ovary syndrome (PCOS) is a disease of the hypothalamus–pituitary–ovarian (HPO) axis affecting about 20% of reproductive women worldwide [5,6]. PCOS is marked by anovulation, increased androgen secretion, and polycystic ovaries [7,8]. The presence of two out of the three characteristics of PCOS is needed to make a clinical diagnosis according to the Rotterdam criteria [9]. Other features include uneven gonadotropin secretion, i.e., increased luteinizing hormone (LH), increased LH:FSH (follicle-stimulating hormone) ratio, low sex hormone-binding globulin (SHBG), and chronic inflammation [10,11].

Endometrial Cancer (EC) is one of the most common malignancies occurring in women, accounting for about 142,000 cases and 42,000 deaths worldwide. Type 1 EC, which is the most common lesion is associated with an excellent prognosis, while Type 2 EC is often high grade and tends to recur [12].

Dumesic et al. suggested that women who were diagnosed with PCOS have a 2.7-fold increased risk of developing endometrial cancer (EC) [13]. Unfortunately, the exact mechanisms that increase the risk of EC in PCOS are unclear. The pathophysiology of the increased risk is thought to involve the exposure of the endometrium to abnormally high levels of estrogen because of anovulation unopposed to progesterone; however, this is uncertain [14].

The gut microbiome plays a pivotal role in modulating normal physiology and immune functions [1]. Dysbiosis of the gut microbiome with altered microbial composition and diversity has been associated with a myriad of disease conditions such as Type 2 Diabetes Mellitus, Inflammatory Bowel Disease, and severe conditions such as cancers [15].

Although PCOS and EC are different diseases, given the increased risk of EC in PCOS, a range of potential mechanisms has been explored as possible mechanisms underpinning the association, but not the possible role of gut microbiota.

Previous studies investigating the gut microbiome in PCOS [16,17,18,19] and EC patients [20] suggest a role for dysbiosis in the etiologies of both conditions. So far, no studies have sought to investigate whether commonalities in gut dysbiosis in PCOS and EC may explain the increased risk of EC in PCOS. However, alterations in alpha and beta diversity of the gut microbiome and the associated intestinal dysfunction have been postulated to play a role in the exacerbation of PCOS [21]. Gut dysbiosis results in abnormal activation of the immune system that interferes with the insulin receptors present in the body, causing hyperinsulinemia, which in turn elevates the secretion of androgens from the ovaries, preventing the formation of normal ovarian follicles [7]. With respect to EC, the most accredited theory with respect to the gut microbiota is that of the activity of the enzyme β-glucuronidase. Previous work by Baker et al. has shown a role for beta-glucuronidase produced by the gut microbiota in the regulation and deconjugation of estrogen into its active form [22]. Hence, with dysbiosis, the alteration in the modulation of this enzyme by the estrobolome could contribute to the development of endometrial hyperplasia and cancer [22].

As we did not identify any previous studies investigating whether commonalities in gut dysbiosis in PCOS and EC may explain the increased risk of EC in PCOS, we set out to perform a scoping review of the literature to determine the extent and nature of published studies on the gut microbiota in, PCOS and EC, and attempt to find any similarities between the composition of the microbiota to determine whether the gut microbiota may contribute to the pathogenesis of EC in those with PCOS.

## 2. Materials and Methods

Institutional review board approval was not required for this study as it did not involve direct contact with patients, and it was a secondary review of primary studies in the literature.

### 2.1. Eligibility Criteria

The published articles reviewed in this study were limited to studies published between the years 2016 to 2022. This was because of the completion of the human microbiome project in 2016, which was a major milestone in enhancing our understanding of the human microbiome. We also limited the study to studies on humans and excluded all studies on animal models to maintain relevance to clinical practice as well as any review articles. The literature search was also limited to studies published in the English language.

Articles had to be focused on the gut microbiota, PCOS, and EC as well as including specific keywords such as ‘gut microbiome’, ‘gut microbiota’, ‘gut dysbiosis’, ‘PCOS’, and ‘endometrial cancer’.

### 2.2. Information Sources

To collect all the relevant published articles, we used two databases: PubMed and Scopus. PubMed is a free search engine to search medicine and biomedical journal literature. It searches several databases and interfaces Medline, directly. This search engine maps user’s search terms to the Medical subject heading (Mesh) and text words in Medline records and then searches [23]. Scopus is an abstract and indexing database with full-text links [24]. The timeline of the articles was filtered in the search engines following our previously outlined eligibility criteria. Two reviewers individually navigated the databases and exported the relevant articles into a spreadsheet, and once data collection was completed, the articles were reviewed once more in a group setting, and duplicates were removed.

### 2.3. Search

The search strategy for PubMed involved using the following combination of keywords such as ‘gut microbiome and pcos’, ‘gut microbiota and pcos’, ‘gut dysbiosis and pcos’, ‘gut microbiome and endometrial cancer’, ‘gut microbiota and endometrial cancer’, ‘gut dysbiosis and endometrial cancer’. The time range was limited to 2016 to 2022.

The search strategy for Scopus involved using the following combination of keywords such as ‘gut microbiome and pcos’, ‘gut microbiota and pcos’, ‘gut dysbiosis and pcos’, ‘gut microbiome and endometrial cancer’, ‘gut microbiota and endometrial cancer’, ‘gut dysbiosis and endometrial cancer’. The time range was limited to 2016 to 2022. Publications were also limited to ‘All open access articles’ and by provided categories into ‘intestinal flora’, ‘polycystic ovary syndrome’, ‘human studies’, ‘endometrial cancer’.

### 2.4. Selection of Sources of Evidence

Following the completion of the data search, articles presented by the databases were split into two halves and assigned to one of two reviewers to be screened individually. Reviewers first screened article titles and if keywords were present the reviewer would then screen the abstract followed by the discussion and conclusion of each study. Data were then extracted from chosen studies and then finally cross-reviewed by both reviewers. Duplicate publications were removed.

### 2.5. Data Charting

The selected articles were read by two reviewers to extract the required data. A table was constructed that included the required variables that were needed to be extracted from each article. Once data charting was completed individually, these data were further reviewed in a group setting.

### 2.6. Data Items

The data extracted included the authors’ names and the study design. Other variables included the ‘study sample size’ (if applicable), which included the number and description of participants in the study; ‘sequencing technique used’ (if applicable), which was the method used for sequencing the bacterial composition obtained from the participants in the study; and ‘results of the study’ (here information regarding the microbial composition or microbial diversity changes in the gut was included as well as any relevant information regarding conclusions made with respect to the gut microbiota).

#### Synthesis of Results

Once data charting was complete, two tables were constructed. One for PCOS and the other for EC. Comparisons of the PCOS and EC data to identify any identify commonalities in the microbial composition and any associated changes were carried out. This comparison was then summarized narratively.

## 3. Results

### 3.1. Selection of Sources of Evidence

**Figure 1:**
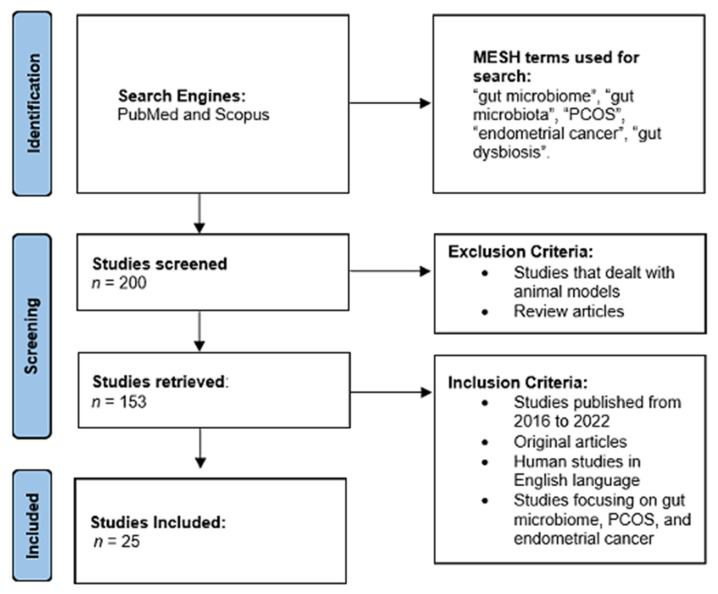
PRISMA Chart detailing the results from the literature search and the criteria applied.

Figure 1 is an illustration of the PRISMA Chart describing the results from the literature search. Following our initial search, we identified 200 results that we then screened. After applying the exclusion criteria, we reduced the list to 153 articles. We then applied our inclusion criteria, which resulted in a list of 25 articles. Of these 25 articles, 23 pertained to gut microbial changes in PCOS and 2 to EC.

### 3.2. Characteristics of Sources of Evidence

The articles (*n* = 25) compiled and charted consist of several different study designs and varied samplings. The included articles were grouped into two main categories, i.e., those that discuss PCOS and others that discuss EC. Descriptive features such as the study authors, sample size, sequencing method, as well as study type pertaining to studies based on both PCOS as well as EC are summarized in Table 1 and Table 2, respectively. The articles were original studies that had an objective relevant to the microbial composition change with regard to PCOS or EC. The charted findings for each of the compiled articles are summarized in Table 1 and Table 2. The results outline the diversity and microbial changes in the gut microbiota pertaining to PCOS and EC.

### 3.3. Synthesis of Results

There were more (23 articles) relevant to PCOS and the microbiome compared to EC articles (2 articles). The frequency of mention of different microbial changes and the modal change that was discovered were noted in both tables. In PCOS, the most frequent mention of a decrease in microbial abundance was in Firmicutes [25,26,27,28] and Prevotellaceae [26,29,30], which was reported in 4 and 3 articles, respectively. Whilst opposingly, a frequency of mention of an increase in abundance in *Bacteroides vulgatus* [31,32], *Escherichia* [31,33], and *Streptococcus* [18,34,35] was present in 2 articles for each apart from *Streptococcus* (3 articles). The microbiome in women with EC, also, demonstrated a decrease in the Firmicutes to Bacteroidetes ratio [36].

Data from both tables regarding microbial changes only shared two common phyla, Bacteroidetes and Firmicutes. Results from the EC articles (1 article) [36] revealed an increase in the Bacteroidetes phylum while, opposingly, results from PCOS articles (2 articles) [26,34], revealed a decrease in the same phylum. This was one piece of evidence of opposing results when comparing PCOS to EC, with other instances of such results prevalent in the PCOS table of results. The phylum Firmicutes was also mentioned in PCOS and EC studies with the results being in agreement as 3 articles [25,26,27] from PCOS showed a decrease similar to the findings in one EC study [37].

There was variability in contradictions in findings pertaining to the similarities in microbial composition change between both diseases.

**Table 1:** Publications on the microbiome in PCOS.

Study Author	Study Type	Sample Groups	Sequencing	Diversity and Microbial Composition Change (Results)
Bo Zeng et al. [29]	Pilot study	9 IR-PCOS (insulin resistant) patients, 8 NIR-PCOS (only PCOS), and 8 healthy controls	16S rRNA	Decrease in the amount of Prevotellaceae in PCOS vs. healthy counterparts, increase of Bacteroidaceae in PCOS patients, and reached its highest level in IR-PCOS patients
Christoph Haudum et al. [38]	Case-Control study	24 patients with PCOS and 19 without PCOS	16S rRNA	Decreased richness in PCOS compared to controls.
Cristina Garcia-Beltran et al. [30]	Randomized Clinical Trials study	23 girls with PCOS that are not obese; 31 age-matched controls	16S ribosomal subunit gene amplicon	Decreased richness in girls with PCOS, more abundance of Family XI, less abundance of family Prevotellaceae, the genus *Prevotella*, and *Senegalimassilia* as compared to controls.
Dong S et al. [37]	Case-Control study	45 patients with PCOS and 37 healthy controls	16S rDNA full-length assembly sequencing technology (16S-FAST)	Decreased richness, increased abundance of *Ruminococcus gnavus*, *Prevotella stercorea*, *Dialister succinatiphilus,* and *Bacteroides fragilis*, decreased abundance of *Christensenellaceae* spp in women with PCOS
Eyupoglu ND et al. [39]	Prospective Observational study	17 overweight/obese patients with PCOS and 15 control women	16S rRNA	Increase in the abundance of Ruminococcaceae in women with PCOS (*p* = 0.006)
Fu Chen et al. [26]	Case-Control study	98 PCOS patients with a normal BMI (PCOS-LB, BMI < 24), 50 PCOS patients with high BMI (PCOS-HB, BMI ≥ 24), and 38 healthy individuals with a normal BMI	16S rRNA	Firmicutes and Actinobacteria were abundant in the healthy group, while Bacteroidetes and Proteobacteria were lower in the PCOS group. PCOS-HB group was featured as a higher abundance of Proteobacteria and *Fusobacteria*. Healthy individuals were featured as higher *Faecalibacterium* and *Prevotella* while lower *Bacteroides* and the PCOS-HB group had a higher abundance of *Bacteroides* and *Megamonas* than the healthy group. Decreased alpha diversity between PCOS and controls as well as a significant difference in beta diversity. PCOS patients have been shown to have a higher abundance of *Catenibacterium*, *Kandleria*, Ruminococcaceae, Bacteroidaceae, *Parabacteroides*, *Clostridium*, *Prevotella*, and *Alistipes* while a lower abundance of Prevotellaceae.
Gulnar Mammadova et al. [40]	Case-Control study	24 lean patients with PCOS A phenotype and 22 BMI-matched healthy women	16 S rDNA V3–V4 region	Erysipelotrichaceae, Proteobacteria, Gammaproteobacteria, Enterobacteriaceae, Planococaceae, Gemmales, and Bacillales were significantly abundant in the PCOS group, while *Clostridium sensu stricto* and *Roseburia* were decreased compared to controls
Hassan S et al. [41]	Case-Control study	19 drug-naive women with PCOS and 20 control women	16S rRNA	Increase in the abundance of Bifidobacteriaceae and decrease in Aerococcaceae and Peptococcaceae in women with PCOS
He F et al. [19]	Case-Control study	14 PCOS patients with insulin resistance (PCOS-IR), 12 PCOS alone (PCOS-NIR), and 10 healthy controls	16 S rDNA V3–V4 fragment	Higher abundance of *Akkermansia* and *Enterococcus* in women with PCOS
Insenser M et al. [42]	Cross-sectional study	15 women with PCOS, 16 non-hyperandrogenic control women, and 15 control men	16S ribosomal DNA	Reduction in β diversity and increase in the abundance of the *Catenibacterium* and *Kandleria* genera in women with PCOS
Jobira B et al. [34]	Prospective, case-control cross-sectional study	37 obese women with PCOS and 21 obese women without PCOS	16S rRNA	Reduced richness, higher relative abundance percent (%RA) of the phyla Actinobacteria (*p* = 0.027), lower Bacteroidetes (*p* = 0.004), but similar Firmicutes and Proteobacteria. PCOS had lower %RA of families Bacteroidaceae (*p* < 0.001) and Porphyromonadaceae (*p* = 0.024) and higher Streptococcaceae (*p* = 0.047).
Lindheim LA-O et al. [43]	Pilot study	24 PCOS patients and 19 healthy controls	16S rRNA	Decrease in the abundance of phylum Tenericutes, ML615J-28, and S24-7 in PCOS women
Li N. et al. [28]	Case-Control study	10 PCOS patients and 10 healthy controls	16S rRNA	The relative abundance of Firmicutes was reduced and the relative abundance of Bacteroidetes was increased in PCOS patients compared with the controls using the fecal samples
Lüll K et al. [18]	Prospective, Case-Control study	102 PCOS women and 201 control women	16S rRNA of V3–V4 regions	Increase in *Paraprevotella*–*Streptococcus* and *Eubacterium ventriosum*–*Bifidobacterium* in women with PCOS
Rui Liu et al. [35]	Cross-sectional study	33 patients with PCOS (12 non-obese/21 obese) and 15 control women (9 non-obese/ 6 obese)	16 S rDNA V3–V4 region	Increased CAGs: *Bacteroides*, *Escherichia*/*Shigella,* and *Streptococcus*. Decreased CAGs: *Akkermansia* and Ruminococcaceae
Torres PJ et al. [44]	Case-Control study	73 women with PCOS, 43 women with PCOM, and 48 healthy controls	16S rRNA	Lower α diversity. Increase in the abundance of *Porphyromonas* spp., *Bacteroides coprophilus*, *Blautia* spp., and *Faecalibacterium prausnitzii*. Decreased abundance of *Anaerococcus* spp., *Odoribacter* spp., *Roseburia* spp., and *Ruminococcus bromii* in women with PCOS.
Weiwei Chu et al. [31]	Case-Control study	14 patients at reproductive age with PCOS and 14 controls	Shotgun metagenomic sequencing	Increased *Parabacteroides merdae*, *Bacteroides fragilis*, and strains of *Escherichia* and *Shigella* in the PCOS group. Increased *Faecalibacterium prausnitzii* in control.
Xinyu Qi et al. [32]	Case-control study	43 healthy control donors and 50 individuals with PCOS were recruited BMI matched to diminish the effect of obesity	Whole-genome shotgun sequencing	Increase in *Bacteroides vulgatus* in PCOS. No significant difference in alpha diversity. Decreased beta diversity in PCOS.
Yuanjiao Liang et al. [27]	Preliminary report	8 obese PCOS (PO group), 10 non-obese PCOS (PN group), and 9 healthy normal-weight women (control) (C group)	16 S rDNA V3–V4 region	Increased *Bacteroides*. Decreased Firmicutes. Decrease in alpha diversity in obese PCOS patients as compared to controls.
Liang Z et al. [33]	Case-Control study	20 women with PCOS (lean PCOS, PL, *n* = 10; overweight PCOS, PO, *n* = 10) and 20 healthy control women (lean control, CL, *n* = 10; overweight control, CO, *n* = 10)	16 S rDNA V3–V4 region	Increase in gamma-aminobutyric acid (GABA)-producing species in PCOS, including *Parabacteroides distasonis*, *Bacteroides fragilis,* and *Escherichia coli*. Decrease in alpha diversity of gut microbiota of the women with PCOS from controls.
Zhang J et al. [45]	Experimental study	38 PCOS patients and 26 control patients	16S rRNA	The abundance of *Faecalibacterium*, *Bifidobacterium,* and *Blautia* was shown to be higher in the control group, while that of *Parabacteroides* and *Clostridium* was in PCOS
Zhou L et al. [46]	Cross-sectional study	60 women with PCOS (30 obese and 30 non-obese) and 41 control women (30 healthy and 11 healthy but obese)	16S rRNA	Decreased abundance of phylum Synergistetes in women with PCOS, *Lactococcus* was the characteristic gut microbiota in NG (non-obese with PCOS), while Coprococcus_2 in OG (obese with PCOS) and decrease in Tenericutes in non-obese women with PCOS.
Zhou L et al. [25]	Case-Control study	18 obese patients with PCOS and 15 obese control women without PCOS	16S rRNA	Decreased ratio of Firmicutes/*Bacteroides* as well as increased abundance of *Fusobacteria* (*p* = 0.022), while a reduced abundance of Tenericutes (*p* = 0.018).

**Table 2:** Publications on the microbiome in Endometrial Cancer.

Study Author	Study Type	Sample Groups	Sequencing	Diversity and Microbial Composition Change (Results)
Adalberto Gonzalez et al. [36]	Case-Control study	8 Patients total (5 female, 3 male): 5 with Lynch syndrome mutation without cancer; 3 with lynch syndrome and cancer (LS-C) (2 with endometrial cancer, 1 with ovarian cancer)	16S ribosomal subunit V3–V4 region	Increased Bacteroidetes (42.2% vs. 28.5%; *p* = 0.068) and Verrucomicrobia (0.644% vs. 0.0007%; *p* = 0.10), and a decreased Firmicutes (48.3% vs. 65.4%; *p* = 0.078) in LS-C patients. LS-C patients had increased *Akkermania* (0.766% vs. 0.001%; *p* = 0.11) and *Bacteroides* (26.6% vs. 17.3%; *p* = 0.44) and decreased *Pseudobutyrvibrio* (0.74% vs. 2.71%; *p* = 0.10), *Enterorhabus* (0.006 vs. 0.07; *p* = 0.18), and *Ruminiclostridium* (0.29 vs. 2.0; *p* = 0.17).
Li C et al. [47]	Prospective, Case-Control study	30 patients with endometrial cancer and 10 healthy controls	16S rRNA	Those with endometrial cancer showed high levels of *Prevotella* and *Pelomonas* associated with a high tumor burden. *Prevotella* in endometrial tissue coupled with high serum d-dimer and Fibrin Degradation Products may be an important factor associated with tumor burden.

## 4. Discussion

Our scoping review identified 25 articles on the gut microbiome in women with PCOS and women with EC, with most (23 articles) of the studies published on PCOS. Although the literature is growing in this field, we did not identify enough published studies, in particular, original research papers, to enable us to investigate whether gut dysbiosis may explain the increased EC risk in PCOS.

Within the studies identified, we were unable to identify any consistent patterns of the microbiome similarly present in studies on women with PCOS compared with women with EC. Although we found that the phylum Firmicutes was similarly decreased in women with PCOS and studies on women with EC, there was, however, significant variability within the studies identified making it highly likely that this may have arisen by chance.

Although we did not identify enough published studies to enable us to investigate whether gut dysbiosis may explain the increased EC risk in PCOS, previous studies on the microbiome in PCOS and EC suggest that it is not unreasonable to investigate the gut microbiome as a possible link between PCOS and EC. The major microbial changes observed in women with PCOS and its consequences are as follows: an increase in *Escherichia* and *Shigella,* which causes an alteration in the short-chain fatty acids, impacting metabolism, immune response, and gut barrier permeability [21,48]; an increase in Prevotellaceae, resulting in a profound and unfavorable inflammatory response to the patient [48,49,50]; and an increase in *Bacteroides vulgates* causing a subsequent reduction in the levels of glycodeoxycholic and tauroursodeoxycholic acid [48].

Boutriq S et al. showed that, normally, estrogen is first conjugated (inactivated) by the liver and this conjugated estrogen is transported to the intestine for its excretion. However, due to dysbiosis of the estrobolome, this conjugated/inactivated estrogen can be converted back to its active form by the process of deconjugation under the influence of certain enzymes produced by the gut microbiota (beta-glucuronidase), leading to high levels of activated estrogen in the blood [20]. Bacterial species responsible for the deconjugation of the conjugated-estrogen complex are Clostridia and Ruminococcacaeae. They are known to influence tumorigenesis by the process of deconjugation [51].

The findings from our scoping review, that the phylum Firmicutes was similarly decreased in women with PCOS and in studies on women with EC, are, however, inconsistent with the gut microbiome underpinning the association between PCOS and EC. This is because obesity, a known risk factor for EC and PCOS, is associated with an increase in the levels of Firmicutes with an increased ratio of Firmicutes to Bacteroidetes [52].

Whilst the strength of this scoping review was in its originality, the low number of publications identified was a limitation. Studies pertaining to the microbiome, specifically, the gut microbiota and its implications in gynecological cancers, are still developing, and this will explain the low number of articles identified, especially those related to the gut microbiome of EC patients. In addition, differences in the patients’ characteristics across studies as well as a low sample size for most of the studies leading to low statistical power may create a bias. These may also explain the differences in findings across studies, which made comparative analysis challenging

The role of an altered gut microbial diversity and composition as one of the mechanisms in the development of EC in those diagnosed with PCOS has hitherto remained unexplored. In this scoping review, we have assessed the existing literature in an attempt to address this pertinent research question. While the literature is growing in this field, we did not identify enough published studies to enable a conclusive answer regarding the potential association to be provided. Although our findings showed that the phylum Firmicutes was similarly decreased in women with PCOS and studies on women with EC, there was, however, significant variability within the studies identified, making it highly likely that this may have arisen by chance. This highlights the need for robust studies aimed at investigating the role of dysbiosis of the gut microbiome in the development of EC and PCOS. Importantly, such studies should investigate the potential role of dysbiosis in stimulating the mechanisms at play in the pathophysiology of EC in patients with PCOS. Furthermore, we speculate that the vaginal microbiota of these patients could also yield important information; although, none of the studies we identified investigated this. We, therefore, highlight this as a gap in the literature and recommend that future studies should incorporate the investigation of both gut and vaginal microbiomes to provide a holistic picture of the extent of dysbiosis in these patients. From a clinical perspective, these studies are important as the potential intervention of reversing dysbiosis via microbiome replacement approaches, whereby the use of probiotics and fecal transplantations could be of value in reducing the development of EC in patients with PCOS. Although data from this scoping review do not address such clinical applicability, the findings enabled us to identify gaps in the literature and areas for future research.

## 5. Conclusions

In conclusion, this scoping review has identified important gaps in the existing literature regarding the gut microbiome in EC and PCOS patients. Our findings indicate a need for more robust studies to address this important research question of the role of dysbiosis in PCOS patients and the link with EC with particular emphasis on elucidating the possible mechanisms involved.

## Data Availability

All the relevant data are contained in the manuscript.

## References

[1] Rosean C.B., Feng T.Y., Azar F.N., Rutkowski M.R. (2019). Impact of the Microbiome on Cancer Progression and Response to Anti-Cancer Therapies. Adv. Cancer Res..

[2] Gopalakrishnan V., Helmink B.A., Spencer C.N., Reuben A., Wargo J.A. (2018). The Influence of the Gut Microbiome on Cancer, Immunity, and Cancer Immunotherapy. Cancer Cell.

[3] Marchesi J.R., Ravel J. (2015). The Vocabulary of Microbiome Research: A Proposal. Microbiome.

[4] Rinninella E., Raoul P., Cintoni M., Franceschi F., Abele G., Miggiano D., Gasbarrini A., Mele M.C. (2019). Microorganisms What Is the Healthy Gut Microbiota Composition? A Changing Ecosystem across Age, Environment, Diet, and Diseases. Microorganisms.

[5] Norman R., Dewailly D., Legro R., Hickey T. (2007). Polycystic ovary syndrome. Lancet.

[6] Escobar-Morreale H.F. (2018). Polycystic Ovary Syndrome: Definition, Aetiology, Diagnosis and Treatment. Nat. Rev. Endocrinol..

[7] Yurtdaş G., Akdevelioğlu Y. (2020). A New Approach to Polycystic Ovary Syndrome: The Gut Microbiota. J. Am. Coll. Nutr..

[8] Yılmaz Ö., Pala H.G., Artunç Ülkümen B. (2017). Comparison of Insulin Sensitivity Levels in Women with PCOS and Women with Regular Menses. Kafkas J. Med. Sci..

[9] Smet M.-E., McLennan A. (2018). Rotterdam Criteria, the End. Australas J. Ultrasound Med..

[10] Hart R., Hickey M., Franks S. (2004). Definitions, Prevalence and Symptoms of Polycystic Ovaries and Polycystic Ovary Syndrome. Best Pract. Res. Clin. Obstet. Gynaecol..

[11] Ehrmann D.A. (2005). Polycystic Ovary Syndrome. N. Engl. J. Med..

[12] Amant F., Moerman P., Neven P., Timmerman D., van Limbergen E., Vergote I. (2005). Endometrial Cancer. Lancet.

[13] Dumesic D.A., Lobo R.A. (2013). Cancer Risk and PCOS. Steroids.

[14] Shafiee M.N., Chapman C., Barrett D., Abu J., Atiomo W. (2013). Reviewing the Molecular Mechanisms Which Increase Endometrial Cancer (EC) Risk in Women with Polycystic Ovarian Syndrome (PCOS): Time for Paradigm Shift?. Gynecol. Oncol..

[15] Weiss G.A., Hennet T. (2017). Mechanisms and Consequences of Intestinal Dysbiosis. Cell. Mol. Life Sci..

[16] Guo Y., Qi Y., Yang X., Zhao L., Wen S., Liu Y., Tang L. (2016). Association between Polycystic Ovary Syndrome and Gut Microbiota. PLoS ONE.

[17] Tremellen K., Pearce K. (2012). Dysbiosis of Gut Microbiota (DOGMA)—A Novel Theory for the Development of Polycystic Ovarian Syndrome. Med. Hypotheses.

[18] Lüll K., Arffman R.K., Sola-Leyva A., Molina N.M., Aasmets O., Herzig K.-H., Plaza-Díaz J., Franks S., Morin-Papunen L., Tapanainen J.S. (2021). The Gut Microbiome in Polycystic Ovary Syndrome and Its Association with Metabolic Traits. J. Clin. Endocrinol. Metab..

[19] He F., Li Y. (2021). The Gut Microbial Composition in Polycystic Ovary Syndrome with Insulin Resistance: Findings from a Normal-weight Population. J. Ovarian Res..

[20] Boutriq S., González-González A., Plaza-Andrades I., Laborda-Illanes A., Sánchez-Alcoholado L., Peralta-Linero J., Domínguez-Recio M.E., Bermejo-Pérez M.J., Lavado-Valenzuela R., Alba E. (2021). Gut and Endometrial Microbiome Dysbiosis: A New Emergent Risk Factor for Endometrial Cancer. J. Pers. Med..

[21] Thackray V.G. (2019). Sex, Microbes, and Polycystic Ovary Syndrome. Trends Endocrinol. Metab..

[22] Baker J.M., Al-Nakkash L., Herbst-Kralovetz M.M. (2017). Estrogen–Gut Microbiome Axis: Physiological and Clinical Implications. Maturitas.

[23] Reza Samadzadeh G., Rigi T., Reza Ganjali A. (2013). Comparison of Four Search Engines and Their Efficacy with Emphasis on Literature Research in Addiction (Prevention and Treatment). Int. J. High. Risk Behav. Addict..

[24] Burnham J.F. (2006). Scopus Database: A Review. Biomed. Digit. Libr..

[25] Zhou L., Ni Z., Yu J., Cheng W., Cai Z., Yu C. (2020). Correlation Between Fecal Metabolomics and Gut Microbiota in Obesity and Polycystic Ovary Syndrome. Front. Endocrinol..

[26] Chen F., Chen Z., Chen M., Chen G., Huang Q., Yang X., Yin H., Chen L., Zhang W., Lin H. (2021). Reduced Stress-Associated FKBP5 DNA Methylation Together with Gut Microbiota Dysbiosis Is Linked with the Progression of Obese PCOS Patients. NPJ Biofilms Microbiomes.

[27] Liang Y., Ming Q., Liang J., Zhang Y., Zhang H., Shen T. (2020). Gut Microbiota Dysbiosis in Polycystic Ovary Syndrome: Association with Obesity—A Preliminary Report. Can. J. Physiol. Pharmacol..

[28] Li N., Li Y., Qian C., Liu Q., Cao W., Ma M., He R., Chen R., Geng R., Liu Y. (2021). Dysbiosis of the Saliva Microbiome in Patients With Polycystic Ovary Syndrome. Front. Cell. Infect. Microbiol.

[29] Zeng B., Lai Z., Sun L., Zhang Z., Yang J., Li Z., Lin J., Zhang Z. (2019). Structural and Functional Profiles of the Gut Microbial Community in Polycystic Ovary Syndrome with Insulin Resistance (IR-PCOS): A Pilot Study. Res. Microbiol..

[30] Garcia-Beltran C., Malpique R., Carbonetto B., González-Torres P., Henares D., Brotons P., Muñoz-Almagro C., López-Bermejo A., de Zegher F., Ibáñez L. (2021). Gut Microbiota in Adolescent Girls with Polycystic Ovary Syndrome: Effects of Randomized Treatments. Pediatr. Obes..

[31] Chu W., Han Q., Xu J., Wang J., Sun Y., Li W., Chen Z.-J., Du Y. (2020). Metagenomic Analysis Identified Microbiome Alterations and Pathological Association between Intestinal Microbiota and Polycystic Ovary Syndrome. Fertil Steril..

[32] Qi X., Yun C., Sun L., Xia J., Wu Q., Wang Y., Wang L., Zhang Y., Liang X., Wang L. (2019). Gut Microbiota–Bile Acid–Interleukin-22 Axis Orchestrates Polycystic Ovary Syndrome. Nat. Med..

[33] Liang Z., Di N., Li L., Yang D. (2021). Gut Microbiota Alterations Reveal Potential Gut–Brain Axis Changes in Polycystic Ovary Syndrome. J. Endocrinol. Investig..

[34] Jobira B., Frank D.N., Pyle L., Silveira L.J., Kelsey M.M., Garcia-Reyes Y., Robertson C.E., Ir D., Nadeau K.J., Cree-Green M. (2020). Obese Adolescents with PCOS Have Altered Biodiversity and Relative Abundance in Gastrointestinal Microbiota. J. Clin. Endocrinol. Metab..

[35] Liu R., Zhang C., Shi Y., Zhang F., Li L., Wang X., Ling Y., Fu H., Dong W., Shen J. (2017). Dysbiosis of Gut Microbiota Associated with Clinical Parameters in Polycystic Ovary Syndrome. Front. Microbiol..

[36] Gonzalez A., Kapila N., Melendez-Rosado J., Liang H., Castro-Pavia F. (2021). An Evaluation of the Fecal Microbiome in Lynch Syndrome. J. Gastrointest. Cancer.

[37] Dong S., Jiao J., Jia S., Li G., Zhang W., Yang K., Wang Z., Liu C., Li D., Wang X. (2021). 16S RDNA Full-Length Assembly Sequencing Technology Analysis of Intestinal Microbiome in Polycystic Ovary Syndrome. Front. Cell. Infect. Microbiol..

[38] Haudum C., Lindheim L., Ascani A., Trummer C., Horvath A., Münzker J., Obermayer-Pietsch B. (2020). Impact of Short-Term Isoflavone Intervention in Polycystic Ovary Syndrome (PCOS) Patients on Microbiota Composition and Metagenomics. Nutrients.

[39] Eyupoglu N.D., Ergunay K., Acikgoz A., Akyon Y., Yilmaz E., Yildiz B.O. (2020). Gut Microbiota and Oral Contraceptive Use in Overweight and Obese Patients with Polycystic Ovary Syndrome. J. Clin. Endocrinol. Metab..

[40] Mammadova G., Ozkul C., Yilmaz Isikhan S., Acikgoz A., Yildiz B.O. (2021). Characterization of Gut Microbiota in Polycystic Ovary Syndrome: Findings from a Lean Population. Eur. J. Clin. Investig..

[41] Hassan S., Kaakinen M.A., Draisma H., Zudina L., Ganie M.A., Rashid A., Balkhiyarova Z., Kiran G.S., Vogazianos P., Shammas C. (2022). Bifidobacterium Is Enriched in Gut Microbiome of Kashmiri Women with Polycystic Ovary Syndrome. Genes.

[42] Insenser M., Murri M., del Campo R., Martínez-García M.Á., Fernández-Durán E., Escobar-Morreale H.F. (2018). Gut Microbiota and the Polycystic Ovary Syndrome: Influence of Sex, Sex Hormones, and Obesity. J. Clin. Endocrinol. Metab..

[43] Lindheim L., Bashir M., Münzker J., Trummer C., Zachhuber V., Leber B., Horvath A., Pieber T.R., Gorkiewicz G., Stadlbauer V. (2017). Alterations in Gut Microbiome Composition and Barrier Function Are Associated with Reproductive and Metabolic Defects in Women with Polycystic Ovary Syndrome (PCOS): A Pilot Study. PLoS ONE.

[44] Torres P.J., Siakowska M., Banaszewska B., Pawelczyk L., Duleba A.J., Kelley S.T., Thackray V.G. (2018). Gut Microbial Diversity in Women with Polycystic Ovary Syndrome Correlates with Hyperandrogenism. J. Clin. Endocrinol. Metab..

[45] Zhang J., Sun Z., Jiang S., Bai X., Ma C., Peng Q., Chen K., Chang H., Fang T., Zhang H. (2019). Probiotic Bifidobacterium Lactis V9 Regulates the Secretion of Sex Hormones in Polycystic Ovary Syndrome Patients through the Gut-Brain Axis. mSystems.

[46] Zhou L., Ni Z., Cheng W., Yu J., Sun S., Zhai D., Yu C., Cai Z. (2020). Characteristic Gut Microbiota and Predicted Metabolic Functions in Women with PCOS. Endocr. Connect..

[47] Li C., Gu Y., He Q., Huang J., Song Y., Wan X., Li Y. (2021). Integrated Analysis of Microbiome and Transcriptome Data Reveals the Interplay between Commensal Bacteria and Fibrin Degradation in Endometrial Cancer. Front. Cell. Infect. Microbiol..

[48] Giampaolino P., Foreste V., di Filippo C., Gallo A., Mercorio A., Serafino P., Improda F.P., Verrazzo P., Zara G., Buonfantino C. (2021). Microbiome and PCOS: State-of-Art and Future Aspects. Int. J. Mol. Sci..

[49] Ley R.E. (2016). Prevotella in the Gut: Choose Carefully. Nat. Rev. Gastroenterol. Hepatol..

[50] Lukens J.R., Gurung P., Vogel P., Johnson G.R., Carter R.A., McGoldrick D.J., Bandi S.R., Calabrese C.R., van de Walle L., Lamkanfi M. (2014). Dietary Modulation of the Microbiome Affects Autoinflammatory Disease. Nature.

[51] Bhatt A.P., Redinbo M.R., Bultman S.J. (2017). The Role of the Microbiome in Cancer Development and Therapy. CA Cancer J. Clin..

[52] Schreurs M.P.H., de Vos van Steenwijk P.J., Romano A., Dieleman S., Werner H.M.J. (2021). How the Gut Microbiome Links to Menopause and Obesity, with Possible Implications for Endometrial Cancer Development. J. Clin. Med..

